# Supplementary material for: Sequential invasion of epithelial and immune cells in Shigellosis
Source: bioRxiv. 2026 Sep 12:2026.09.11.751053. Preprint. [Version 1] doi: 10.64898/2026.09.11.751053 (PMC13622684; doi:10.64898/2026.09.11.751053)
Supplement: Supplementary file 1 [file NIHPP2026.09.11.751053V1-supplement-1.pdf]

bioRxiv preprint doi: https://doi.org/10.64898/2026.09.11.751053; this version posted September 12, 2026. The copyright holder for this preprint (which was not certified by peer review) is the author/funder, who has granted bioRxiv a license to display the preprint in perpetuity. It is made available under a CC-BY 4.0 International license.

Supplementary Files

Supplementary Figures:

Supplementary Fig 1. Image analysis of the infected colonic mucosa.

Supplementary Fig. 2. Cellular infection in Lamina Propria.

Supplementary Tables:

Supplementary Table 1. Annotation of DeSeq2 analysis using the C8 data base.

bioRxiv preprint doi: https://doi.org/10.64898/2026.09.11.751053; this version posted September 12, 2026. The copyright holder for this preprint (which was not certified by peer review) is the author/funder, who has granted bioRxiv a license to display the preprint in perpetuity. It is made available under a CC-BY 4.0 International license.

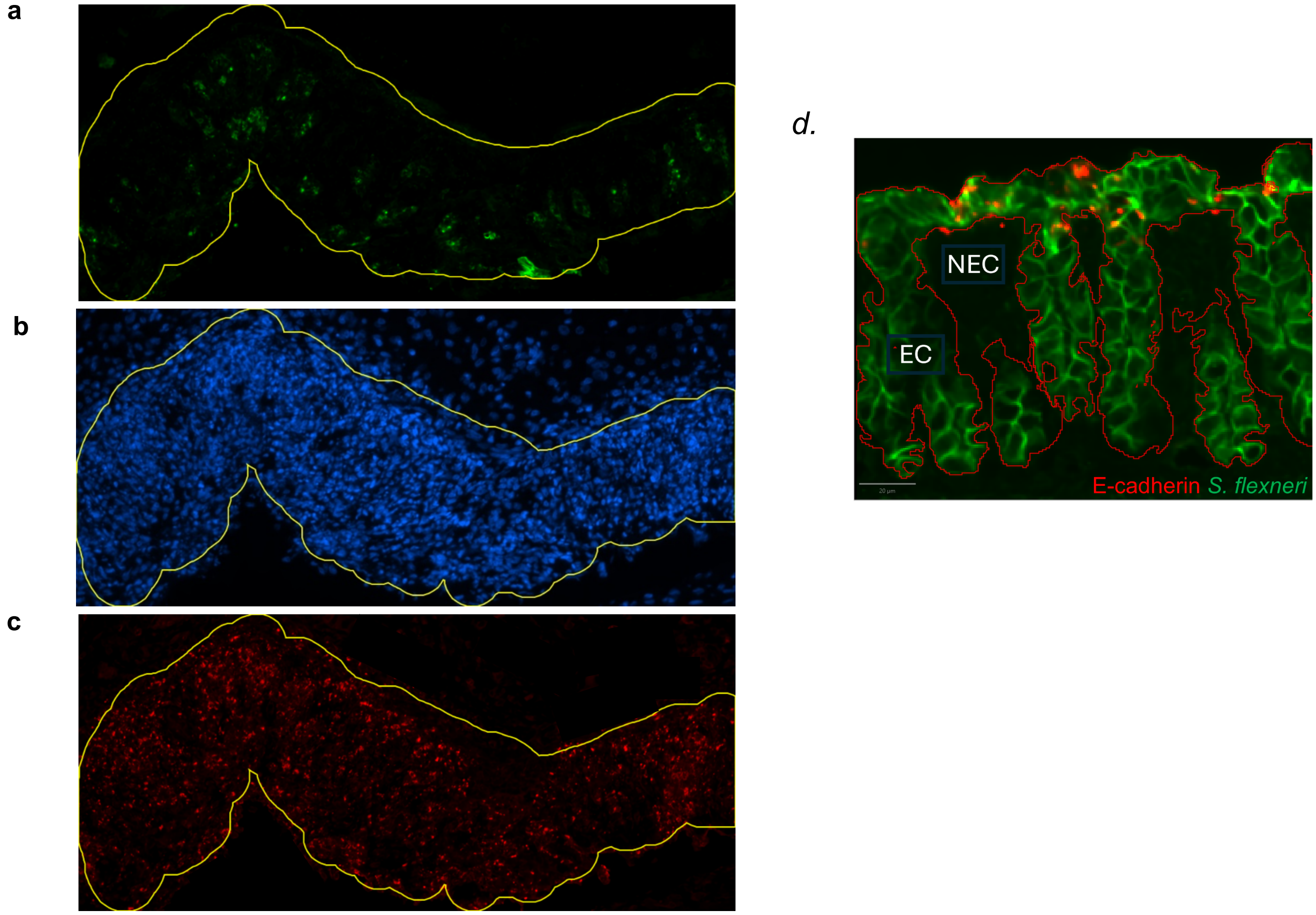

Supplementary Fig. 1. Image analysis methods in the infant rabbit model of bacillary dysentery. Representative fluorescence images illustrating the bacterial quantification pipeline applied to infected colon tissue collected at 24 hpi. **(a)** DAPI (blue) nuclear staining, which is combined with **(b)** E-cadherin (ECAD; green) staining of the intestinal epithelium, which is largely degraded by 24 hpi residual ECAD signal to delineate and create a mask of the mucosal region and measure its corresponding area. **(c)** Bacterial signal (red) in a separate channel. **(d)** Compartment based estimation of bacterial counts within EC and NEC region of Mucosa in infections across time.

bioRxiv preprint doi: https://doi.org/10.64898/2026.09.11.751053; this version posted September 12, 2026. The copyright holder for this preprint (which was not certified by peer review) is the author/funder, who has granted bioRxiv a license to display the preprint in perpetuity. It is made available under a CC-BY 4.0 International license.

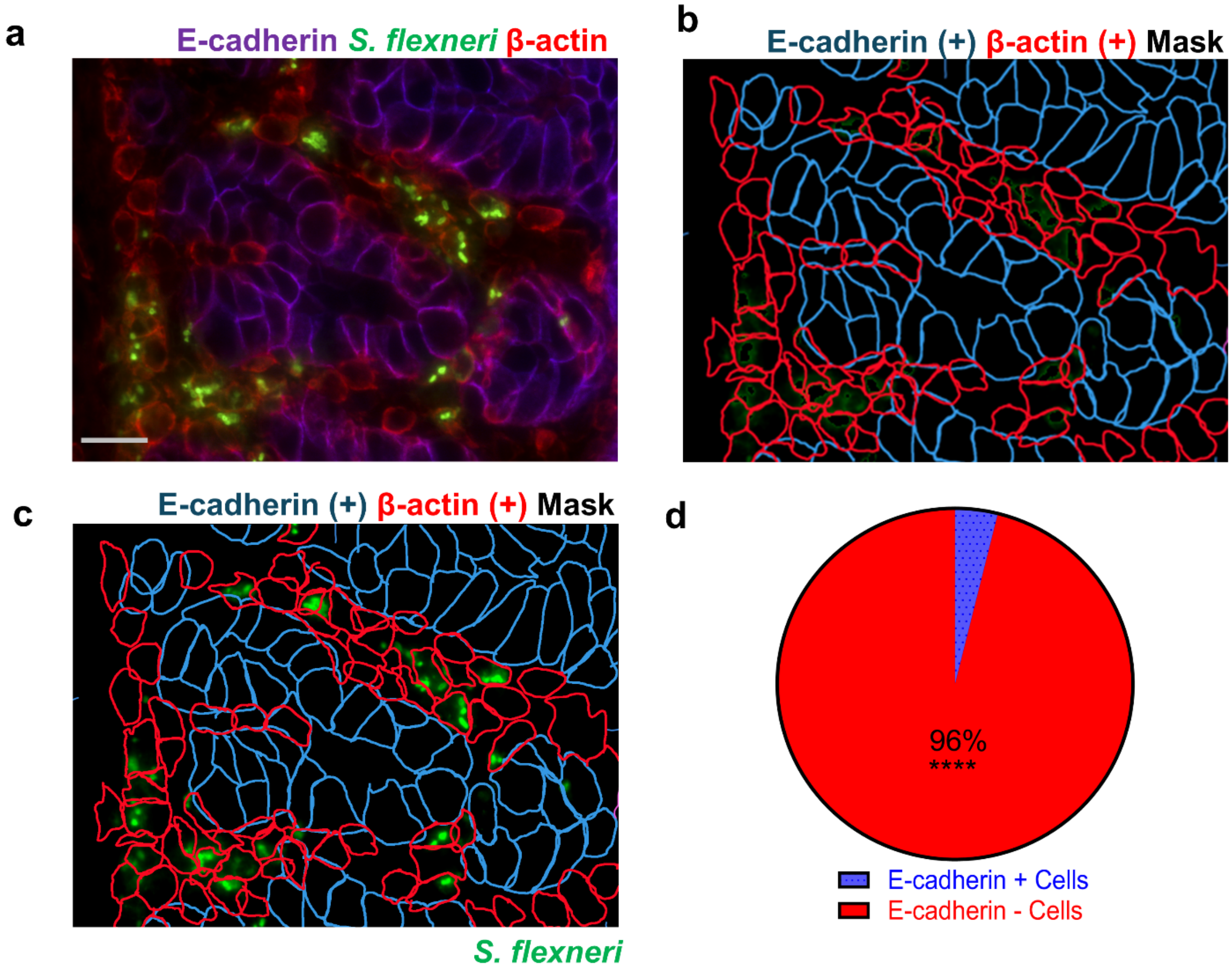

Supplementary Fig. 2. Cellular infection in the lamina propria. **(a)** Representative image of a colonic section at 24 hpi, immuno-stained for E-cadherin (purple), *S. flexneri* (green) and β-actin (red). Scale bar, 50 µm. **(b)** Segmentation mask distinguishing E-cadherin-positive cells (blue outlines) from E-cadherin-negative, β-actin-positive cells (red outlines). **(c)** Overlay of the mask in (b) with the *S. flexneri* channel (green). **(d)** Percentage of infected cells that were E-cadherin-positive (epithelial, blue) or E-cadherin-negative (lamina propria, red), quantified from (c) Percentage of infected cells that were E-cadherin-positive (epithelial) or E-cadherin-negative (lamina propria), quantified from (**c**). n=20 imaging fields; individual fields were treated as replicates. Statistics: Wilcoxon matched-pairs signed-rank test, median difference 47.0%, ****P < 0.0001.

bioRxiv preprint doi: https://doi.org/10.64898/2026.09.11.751053; this version posted September 12, 2026. The copyright holder for this preprint (which was not certified by peer review) is the author/funder, who has granted bioRxiv a license to display the preprint in perpetuity. It is made available under a CC-BY 4.0 International license.

Supplementary Table 1. Annotation of rlog normalized reads analysed using the C8 data base.
Conditions: mock-treated samples (PBS, n=5) and, infected samples (WT n=5). Values are on the rlog scale.

Name	24PBS1	8PBS2	24PBS2	8PBS3	24PBS3	24WT1	24WT2	24WT4	24WT5	24WT3
LGR5	10.4808	10.4398	10.5307	11.0022	10.6141	9.9503	9.3003	9.4542	9.6573	9.6451
AXIN2	10.0094	10.0258	10.0071	9.6753	10.0236	9.5749	9.7211	9.519	9.3698	9.6868
PLAGL2	9.7223	9.9553	9.832	9.9407	9.9892	9.1239	9.3287	9.2564	9.4012	9.2411
SOX9	9.6354	9.6661	9.5036	9.8094	9.4281	9.3159	8.8733	9.3796	9.2537	9.0337
LYZ	19.0603	18.3632	19.1155	18.5928	18.9268	17.6449	16.9699	17.3936	17.9874	17.6669
WNT3	1.3204	1.4754	1.4038	1.5367	1.7135	1.3274	1.3986	1.527	1.3208	1.3601
ATOH1	10.1693	10.0738	10.0098	10.2349	9.8475	9.3139	8.3507	9.1681	9.2782	9.2041
B3GNT6	9.7459	10.1132	9.6737	10.1756	9.8122	9.6049	8.9396	9.5142	9.8305	9.2127
CLCA1	12.9814	13.3616	13.1456	13.6118	13.0618	12.0627	11.3999	11.9722	12.5569	11.8426
SLC37A1	10.6453	10.7258	10.5639	10.658	10.5388	10.3669	10.0968	10.3736	10.1697	10.1882
CLDN8	11.515	11.4798	11.1852	11.6913	11.617	10.213	9.3919	10.356	10.5537	10.3019
AADAC	9.6469	9.5418	9.6138	9.5266	9.5305	8.2829	7.9037	8.3994	9.0206	8.3522
SLC26A3	10.6964	11.1913	11.681	11.178	11.5254	10.1882	8.3306	8.8989	9.7715	9.4657
RNF186	8.9916	8.464	8.7559	8.4351	8.9163	6.8108	6.1156	6.9427	7.1473	7.2974
CSF3R	6.4618	6.2879	6.0108	6.5777	6.1086	8.8447	10.2148	10.3696	10.0846	9.1945
LCN2	7.774	7.792	7.7663	8.1684	7.7528	11.8265	13.0889	13.3101	12.672	12.0071
LTF	6.7501	6.7187	6.9455	6.8608	6.8812	9.5811	10.3388	9.9578	10.2968	10.3442
CAMP	6.4612	6.5325	6.41	6.4658	6.4045	9.9865	11.4167	11.4401	11.19	10.5415
MMP9	5.8787	6.6783	5.9773	6.2661	5.654	8.9075	9.7074	10.1244	9.8179	8.4893
CD14	7.6347	7.532	7.2952	7.5087	7.2253	9.6041	10.3711	10.2643	10.1178	9.6819
OLR1	5.6459	5.5868	5.8679	5.9746	5.6632	8.1038	8.8778	9.2557	8.6933	8.4287
CD68	9.8989	10.1939	9.6091	9.7433	9.8925	10.1474	10.2514	10.8252	10.3318	10.158
CCL2	6.4282	6.4269	6.6739	7.2035	6.472	10.8677	11.1709	10.644	8.8962	10.0971
CSF1R	9.1063	9.0029	8.7587	9.0239	8.7481	9.2358	9.8502	10.1657	10.3145	9.2795
TNF	5.0378	5.0912	5.0742	5.1913	4.8108	7.7998	8.0709	7.9443	7.0864	7.2698
IL1B	6.9959	6.8756	6.9364	7.1733	6.8522	10.9771	11.8773	11.5888	10.4826	11.0902
CXCL10	7.6931	7.3148	7.1403	7.8997	6.645	9.9543	10.3107	10.2425	9.272	9.253
MRC1	9.8329	9.5812	9.7616	9.7572	9.6296	11.0126	11.8127	11.3195	12.3747	11.3546
MARCO	4.945	4.9319	4.8673	5.0276	4.7157	6.3611	7.6036	7.3935	7.3041	5.7607
LYVE1	7.8789	7.7222	7.8551	7.8972	8.076	8.3847	8.6423	8.4843	8.5811	8.481
CD86	5.8482	5.5461	5.435	5.3701	5.0738	5.5529	5.7217	5.6011	5.4564	5.3651
IL6	6.2741	6.1931	6.1349	6.2464	6.1253	11.3665	11.4421	10.7825	8.7439	10.7129
CD163	8.1138	8.0266	7.9764	8.2299	8.0475	9.4916	9.8978	9.9918	10.0415	9.4906
ARG1	5.4575	5.1812	5.4172	5.7678	5.2045	7.5947	8.6825	8.6452	8.6212	7.7368
CD31	10.1979	10.3201	10.1531	10.3668	10.1745	11.1781	11.6335	11.6653	11.6027	11.0925
F3	8.7844	8.4195	8.4348	8.5193	8.5817	11.2963	11.2388	11.9931	10.6581	11.2161
